# Role of Oxidative Stress in Ocular Diseases: A Balancing Act

**DOI:** 10.3390/metabo13020187

**Published:** 2023-01-27

**Authors:** Daisy Y. Shu, Suman Chaudhary, Kin-Sang Cho, Anton Lennikov, William P. Miller, David C. Thorn, Menglu Yang, Tina B. McKay

**Affiliations:** 1 Department of Ophthalmology, Schepens Eye Research Institute of Mass Eye and Ear, Harvard Medical School, Boston, MA 02114, USA; 2 Department of Chemistry and Chemical Biology, Harvard University, Cambridge, MA 02138, USA; 3 Department of Anesthesia, Critical Care and Pain Medicine, Massachusetts General Hospital, Harvard Medical School, Boston, MA 02114, USA

**Keywords:** reactive oxygen species, mitochondrial dysfunction, cornea, retina, age-related macular degeneration, keratoconus, dry eye disease, cataract, proliferative vitreoretinopathy, glaucoma, epithelial–mesenchymal transition

## Abstract

Redox homeostasis is a delicate balancing act of maintaining appropriate levels of antioxidant defense mechanisms and reactive oxidizing oxygen and nitrogen species. Any disruption of this balance leads to oxidative stress, which is a key pathogenic factor in several ocular diseases. In this review, we present the current evidence for oxidative stress and mitochondrial dysfunction in conditions affecting both the anterior segment (e.g., dry eye disease, keratoconus, cataract) and posterior segment (age-related macular degeneration, proliferative vitreoretinopathy, diabetic retinopathy, glaucoma) of the human eye. We posit that further development of therapeutic interventions to promote pro-regenerative responses and maintenance of the redox balance may delay or prevent the progression of these major ocular pathologies. Continued efforts in this field will not only yield a better understanding of the molecular mechanisms underlying the pathogenesis of ocular diseases but also enable the identification of novel druggable redox targets and antioxidant therapies.

## 1. Introduction

Aging is a major risk factor for many diseases, including cancer, cardiovascular disease, and neurodegeneration [1,2]. As we age, our increased susceptibility to disease may partially be attributed to genetic and epigenetic changes that develop over time with continual environmental exposure and damage from endogenous and exogenous reactive oxygen species (ROS). Inept pro-reparative responses and telomere shortening are central to the underlying mechanisms involved in aging [3]. It is thought that the biological age of an organism is largely dependent on the body’s ability to manage internal and external stress at the cellular level, which may lead to cell dysfunction if left uncontrolled. While the gradual deterioration in genomic stability and cellular senescence are inevitable with aging, mutations in mitochondrial DNA (mtDNA), altered proteostasis, and nutrient-sensing also contribute to disrupted bioenergetics, which influences tissue and organismal survival [4].

The human eye is not resistant to processes associated with aging. At the front of the eye, the cornea and lens are particularly susceptible to oxidative stress that can be attributed to direct exposure to ultraviolet (UV) light emitted by the sun. As a highly metabolic tissue, the retina is also at increased risk of age-associated processes that contribute to increased oxidative stress and neurodegeneration. Damage to these cell layers may cause deficits in visual acuity or progressive vision loss and significantly affect mobility and quality of life.

In this review, we introduce each pathological condition affecting the major tissues within the human eye and highlight the role of oxidative stress in their underlying disease etiologies. Understanding the conserved mechanisms involved in the disruption of the cellular redox balance may aid in developing targeted interventions to prevent or delay the progression of the major ocular pathologies associated with oxidative stress.

## 2. Sources of Oxidative Stress

**Figure 1:**
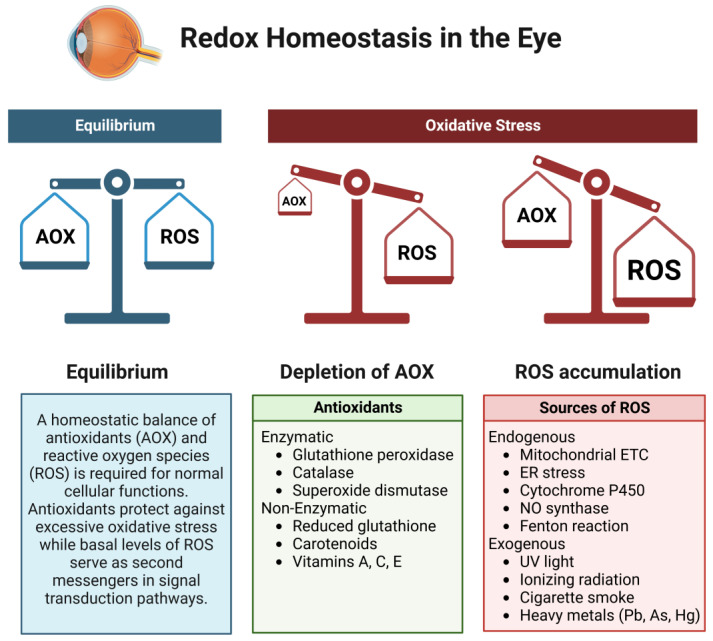
Redox homeostasis in the eye. A homeostatic balance of antioxidants and reactive oxygen species (ROS) is required for the healthy functioning of ocular tissues. An imbalance of antioxidants (AOX) and ROS through either the depletion of antioxidants or excessive ROS accumulation will result in oxidative stress and drive subsequent disease progression. Antioxidants are categorized as either enzymatic or non-enzymatic. ROS can be derived from either endogenous or exogenous sources. Examples of heavy metals include lead (Pb), arsenic (As), and mercury (Hg).

A homeostatic balance exists between the production and quenching of ROS produced during normal physiological processes (Figure 1). The intracellular compartments of a cell maintain a reducing environment with a high abundance of glutathione (GSH) to prevent oxidative damage. The maintenance of this redox balance is essential in permitting downstream signaling processes that rely upon an oxidative state to activate secondary messengers. Of the sources of ROS, oxidative phosphorylation within the electron transport chain (ETC) in mitochondria is the major contributor to superoxide production, which may be converted to hydrogen peroxide via the catalytic activity of superoxide dismutase (SOD). Mitochondria perform essential functions within a cell, serving as primary energy generators in the form of ATP. Somatic mutations in mtDNA may develop gradually over time with age, leading to decreased mitochondrial function and energy output [5]. Mitochondria are thought to acquire mutations at a rate of 10–100X more often than mutations in the nuclear genome [6] due to the increased oxidative environment within the mitochondrion, the absence of protective histones for mtDNA, and the minimal proofreading capabilities of DNA polymerase γ that contribute to lower replication fidelity [7,8].

The metabolism of oxygen produces a low level of ROS in a beneficial, physiological process. Under normal conditions, the ETC promotes ATP synthesis by maintaining a voltage gradient across the inner mitochondrial membrane (ΔΨ_m_) that drives ATP-synthase. Oxygen plays a critical role in this process by acting as the final electron acceptor, serving as a nucleophile for two protons to generate water. However, when ΔΨ_m_ is in excess, oxygen can also be partially reduced to form superoxide (O_2_^·−^), which can then be protonated to form other reactive species, including hydrogen peroxide (H_2_O_2_) and the hydroxyl radical (^·^OH). The oxidative phosphorylation pathway is executed by a series of protein complexes (complexes I–IV) embedded in the mitochondrial membrane [9]. Nicotinamide adenine dinucleotide (NADH2) and flavin adenine dinucleotide (FADH2) donate electrons that are transported between these protein complexes, coupled with the pumping of hydrogen ions across the mitochondrial membrane [9]. Electron leakage from complex I and complex III will react with oxygen and form ROS, including hydrogen peroxide, hydroxyl radicals, and other reactive oxygen and nitrogen species [10,11]. Other non-mitochondrial sources of free radicals are the nitric oxide synthase reaction and cytochrome P450 system, among others [12].

Excess intracellular ROS production can lead to the oxidation of DNA, proteins, lipids, and metabolites, thereby leading to possible disruptions in gene and protein expression patterns, protein aggregation, and cellular dysfunction. Endogenous ROS may also promote the generation of lipid hydroperoxides that function as key intermediates in ROS-mediated processes. The major biomarkers of oxidative stress reported in studies of the eye include malondialdehyde (MDA) and 4-hydroxynonenal (4-HNE), which are byproducts of lipid peroxidation mediated via hydrogen peroxide, superoxide, or other ROS (Table 1). Cyclopentenone prostaglandins are also important mediators involved in lipid peroxidation that have been found to promote ROS production and cytotoxicity.

The cellular antioxidant defense system in most cell types consists of non-enzymatic and enzymatic components responsible for scavenging free radicals and non-radical oxidants [24]. Examples of intracellular non-enzymatic protective mechanisms against excess ROS are metal-binding proteins (albumin, ferritin, lactoferrin), retinol (Vitamin A), ascorbic acid (Vitamin C), tocopherol (Vitamin E), coenzyme Q10, and polyamines (spermine, spermidine, putrescine). The enzymatic protective mechanisms responsible for reacting with ROS include superoxide dismutase (SOD), catalase, peroxiredoxins, glutathione peroxidase, and glutathione reductase, among others. There are three main isoforms of SOD, characterized by their cellular localization: cytosolic (SOD1 (CuZn-SOD)), mitochondrial (SOD2 (Mn-SOD)), and extracellular (SOD3 (EC-CuZn-SOD)). Compared to other tissues, the eye has lower levels of SOD proteins, with the highest distribution present within the retina [25]. The transcription factor nuclear factor erythroid-related factor 2 (Nrf2) is another crucial element of the cellular antioxidant response [26,27]. Upon being activated by a change in the redox balance, it binds to enhancer regions in the promoters of antioxidant genes, known as antioxidant response elements, to facilitate their transcription and generate pools of antioxidant proteins to combat cellular ROS.

Arachidonic acid (ARA) and its metabolites play a crucial role in inflammation and the regulation of oxidative stress. While oxidative stress can enhance ARA mobilization [28], ARA itself can potentiate or, in some cases, confer resistance to oxidative stress. This 20-carbon chain fatty acid belongs to the group of omega-6 (n-6) polyunsaturated fatty acids (PUFAs). In its esterified form, ARA is found covalently bound to membrane phospholipids, enabling the fluidity and flexibility of cell membranes. The stimulation of specific cell-surface receptors activates phospholipase A_2_, triggering the release of the free form of ARA from the cell membrane during inflammation. ARA is then metabolized by enzymes such as cyclooxygenases, lipoxygenases, and cytochrome P450 to generate a spectrum of inflammatory bioactive mediators, including prostaglandins, leukotrienes, and lipoxins [29].

While the role of ARA and oxidative stress has not been well studied in ocular tissues, we can glean evidence of this relationship from other fields. During lipoperoxidation, fatty acids within membrane phospholipids undergo oxidative modification, altering membrane fluidity and resulting in dysregulated cellular signaling and aberrant protein structures [28]. The repair of these phospholipids requires the selective cleavage of peroxidized fatty acid residues and their replacement by native fatty acids. This process can enhance phospholipase A_2_ activity, which facilitates ARA mobilization, leading to enhanced inflammatory signaling [30]. In adult cardiac fibroblasts, hydrogen peroxide was found to activate Nox4 through phospholipase A_2_-dependent ARA production [31]. While oxidative stress can mobilize ARA through Ca^2+^-dependent and Ca^2+^-independent phospholipase A_2_ mechanisms, free ARA can also be generated by alternative, phospholipase A_2_-independent pathways, such as through the inhibition of fatty acid reacylation. For example, the exposure of rat alveolar macrophages [32] and vascular smooth muscle cells [33] to hydrogen peroxide resulted in the impairment of fatty acid esterification and the subsequent accumulation of free ARA.

Conversely, ARA itself can activate key oxidative stress molecules. In HeLa cells, ARA was found to induce the interaction of the p67^phox^-Rac complex with Nox2, leading to the production of superoxide [34]. Intriguingly, in hippocampal slices, ARA has been shown to exhibit neuroprotective effects by defending against oxidative stress through the enhancement of the internal antioxidant system [35]. Further research is required to explore the precise relationship between ARA and oxidative stress in ocular diseases.

## 3. Cornea

The cornea is a transparent avascular tissue layer of about 500 μm thickness located at the front of the eye [36]. The cornea is composed of three primary cellular layers: a stratified epithelium, stromal keratocytes, and a single-layered endothelium. The corneal epithelium forms an external cellular surface, which acts as the frontline defense against environmental pathogens, chemical irritants, and abrasive injury. Corneal epithelial cells are exposed to high concentrations of oxygen and UV radiation and thus require powerful antioxidant defenses to prevent oxidative damage. Within the corneal stroma, corneal keratocytes reside in, secrete, and assemble the surrounding extracellular matrix. The bulk mass of the cornea is attributed to the extracellular matrix of the stroma, primarily composed of collagen types I and V and the proteoglycans keratocan, decorin, lumican, and mimecan, which are important in limiting the collagen fibril diameter and maintaining tissue transparency [37]. There are also a number of resident immune cells that have been detected within the uninjured cornea, including macrophages, dendritic cells, and a small population of γδ T cells [38]. Here, we discuss the role of elevated oxidative stress in two of the most common pathologies affecting the cornea, dry eye disease and keratoconus (KC).

### 3.1. Dry Eye Disease

Dry eye is a multifactorial disease characterized by ocular dryness, discomfort, pain, and tear film instability. Current evidence suggests that ROS generated from environmental factors, such as UV radiation and pollution, contribute to the pathogenesis of dry eye disease [39,40]. These findings correlate with a strong association between dry eye and aging, as excess ROS accumulates with older age [41]. Molecular markers of oxidative stress, such as 8-hydroxy-2-deoxyguanosine (8-OHdG), 4-HNE, and MDA, have been observed in a blink-suppressed dry eye model [42]. 4-HNE and another biomarker of oxidative stress, hexanoyl-lysine, was found to be significantly higher in the conjunctiva of patients with Sjögren’s syndrome and dry eye compared to healthy controls [43]. Moreover, in mice with compromised antioxidant capacity induced by the knockout of SOD-1, atrophy of the lacrimal gland occurs, leading to decreased tear production and eventually dry eye [44]. This evidence supports an association between oxidative stress and dry eye disease in animal models and human clinical populations.

The tear film is a thin layer of liquid covering the corneal surface and conjunctiva, which comprises a lipid layer, an aqueous layer, and a mucous layer. The lipid layer is secreted by the meibomian gland located in the eyelids; the lacrimal gland contributes the bulk of the aqueous layer, and the mucous layer is made up of mucin secreted by conjunctival goblet cells and corneal epithelial cells. Disturbances to any of the three layers will lead to dry eye disease. The current literature on ROS in dry eye disease focuses primarily on the dysfunction of the lacrimal and meibomian glands. Uchino et al. observed lacrimal gland damage and decreased tear production in a tetracyclic-mev-1 conditional transgenic mouse model [45]. In this model, the mev-1 mutant has a genetic dysfunction in complex II of the mitochondrial ETC, leading to the overproduction of prooxidants by the mitochondria. Compared to the wild type, mice carrying the mutation showed increased mRNA expression of inflammatory markers, including tumor necrosis factor (TNF-α), interleukin (IL)-6, IL-1β, and interferon (IFN)-γ, as well as higher infiltration of CD4+ T cells, CD8+ T cells, B cells, and macrophages. Proinflammatory cytokines such as TNF-α have the capacity to produce ROS [46], further potentiating oxidative stress and perpetuating the vicious cycle of ROS accumulation. Damage to the normal structure of the acini was also present in the mutant mice, indicating the role of oxidative stress in causing deficient tear production by directly damaging the lacrimal gland [45].

In addition to direct structural damage, oxidative stress may disrupt the neural reflex arc of tear secretion. The cornea and conjunctiva are innervated by sensory nerve endings originating from the trigeminal ganglia. When the sensory nerve is stimulated, it conducts the signal to the central nervous system, activating the efferent parasympathetic and sympathetic nerves that innervate the acini of the lacrimal gland [47]. Prooxidants on the ocular surface damage the myelin of the afferent sensory nerve, causing reduced signal transmission to the lacrimal gland, which in turn leads to decreased tear secretion [40].

The meibomian glands are sebaceous glands located inside the tarsal plates of both the upper and lower eyelids, with openings at the rims of the eyelids. They secrete meibum, which forms the lipid layer of the tear film. The instability of the lipid layer is known as meibomian gland dysfunction, which is a common cause of evaporative dry eye disease [48]. Meibomian gland dysfunction was observed in SOD1 knockout mice, whereby large lipid droplets were found inside meibomian glands, along with increased inflammatory markers (IL-6 and TNF-α) and the infiltration of CD45+ leukocytes [49]. These data indicate that a deficiency in antioxidant capacity may induce inflammation and structural damage to the meibomian gland.

Over the years, clinicians have attempted to treat dry eye disease with antioxidative agents, including Vitamin B12, iodide iontophoresis, and PUFAs. Patients using hyaluronic acid eye drops supplemented with Vitamin B12 reported significantly reduced levels of prooxidant markers in the tear film with longer tear film breakup times and lower Ocular Surface Disease Index scores [50]. It is noteworthy to mention that this study did not include a hyaluronic acid-only group, and thus, the beneficial effects of additional Vitamin B12 are debatable. Iodide is a reducing agent that can neutralize oxidants. Dry eye patients treated with iodide iontophoresis demonstrated significant improvement in dry eye symptoms and clinical evaluation compared to the control group, where participants were given iodide without an electrical current [51]. More recent studies have focused on the use of PUFAs, mainly omega-3 FAs, such as docosahexaenoic acid (DHA) and eicosapentaenoic acid (EPA). PUFAs are fatty acids in which the hydrocarbon chain contains two or more double bonds. These double bonds enable PUFAs to better neutralize ROS. However, according to a recent review of 34 randomized controlled trials involving more than 4314 adult participants, omega-3 FA intervention showed little to no beneficial effect on the symptomatic control of dry eye disease compared to placebo [52]. Therefore, further research is required to optimize the efficacy and route of administration of antioxidants in treating dry eye disease.

A recent result showed that melatonin, a hormone secreted by the pineal gland, protects corneal epithelial cells in a dry eye disease model [53]. The role of different hormone levels in the development of dry eye has been extensively discussed, including sex hormones [54,55], oxytocin [56], growth hormones [57], and many more. These recent studies provide new ideas for future research to investigate the role of different hormones in the maintenance of the ocular surface.

Moreover, further mechanistic research is necessary to determine the source of prooxidants observed in dry eye disease, as well as how dry eye disease may be induced by uncontrolled oxidative stress.

### 3.2. Altered Metabolism and Bioenergetics in Keratoconus

KC is a corneal ectasia that affects about 1 per 500 to 2000 people worldwide, depending on the geographical region [58]. KC is characterized by a thinning of the corneal stroma that leads to the protrusion of the central cornea, the development of severe astigmatism, and visual deficits. In terms of pathophysiology, growing evidence suggests that oxidative stress may play an important role in the underlying etiology of KC [59,60,61,62]. Elevated lipid peroxidation, levels of MDA, reactive oxygen and nitrogen species, and lower total antioxidant capacity have all been associated with KC [61]. Markers of oxidative stress have been detected in the tears [63,64], sera [65], stromal fibroblasts [66], and corneal tissue [67,68,69] of KC patients. KC is often diagnosed in adolescence and early adulthood and generally stabilizes in midlife [70]. Hence, elevated oxidative stress in the cornea is likely not a result of aging or cumulative DNA damage [71] but rather thought to be partly attributed to acquired defects in mitochondrial function or ROS-scavenging abilities, leading to broad cellular stress in the corneal stroma, decreased collagen expression, keratocyte loss, and, ultimately, corneal thinning [72]. Mutations in mitochondrial-associated genes and reduced levels of SOD1 have been associated with familial and sporadic KC [73,74,75,76,77,78], though SOD1 mutations do not appear to be a universal biomarker of KC in all clinical populations [79,80,81,82]. As recently reviewed, other gene variants associated with KC include lysyl oxidase, collagen types IV and V, and inner mitochondrial membrane peptidase [83].

Elevated proinflammatory factors, including IL-6, TNF-α, and matrix metalloproteinase (MMP)-9, have been detected in KC tears, with higher levels correlating with increased KC severity [84]. Untargeted mass spectrometry analysis of human serum from a small case–control study revealed increased dehydroepiandrosterone sulfate and prostaglandins in patients with KC compared to age-matched controls [85], which is consistent with a study by our group [86] and others [87,88] associating altered hormone levels or their receptors with KC. Growing evidence supports the hypothesis that there is a systemic component involved in KC pathogenesis upstream of the endogenous oxidative stress observed in the cornea [89,90].

In terms of functional differences, KC-derived corneal fibroblasts deposit a thinner extracellular matrix with higher profibrotic collagen type III deposition and lower collagen type I compared to controls [91,92]. Stem cells derived from the corneoscleral rim likewise display decreased proliferative ability and elevated α-smooth muscle actin (α-SMA) expression in KC-derived spheroids [93]. Collectively, these in vitro findings have been consistent with the phenotype of moderate to severe KC in vivo [94], suggesting that the genetic or epigenetic features of KC are retained in early passages of primary corneal stromal cells.

A recurring theme of the underlying KC pathophysiology is the presence of altered cellular metabolism and bioenergetics. A metabolomic study of isolated stromal tissue from patients with KC revealed moderate differences in metabolite levels associated with fatty acid metabolism, the tricarboxylic acid (TCA) cycle, and arginine and proline metabolism [95]. Metabolic differences have also been identified in KC-derived corneal fibroblasts, including differential cytosolic levels of metabolites important in glucose metabolism, lower arginine levels, and increased lactate production compared to controls [66]. We have found that treatment with the antioxidant quercetin modulates steady-state levels of metabolites involved in glycolysis and the TCA cycle and reduces lactate production in corneal fibroblasts [96]. Quercetin treatment also appears to possess antifibrotic properties characterized by decreased collagen type III and α-SMA expression in vitro [97] and the blunting of corneal scarring in vivo [98].

Riboflavin-mediated crosslinking is an FDA-approved treatment for KC that also promotes lower lactate/malate levels in tears [99] and reduced lactate production by corneal fibroblasts [100], providing a degree of stabilization to the metabolic phenotype observed in KC, in addition to stiffening the collagen matrix to blunt progressive thinning. Moreover, basal arginase activity and hydroxyproline levels have been found to be lower in KC-derived keratocytes [101], correlating with a reduction in collagen deposition associated with these cells [102]. Our group has found that arginine supplementation promotes a modest increase in collagen secretion by KC-derived corneal fibroblasts [103], suggesting that exogenous arginine may modulate certain deficiencies in procollagen building blocks and stimulate matrix production at the cellular level. Further investigations of antioxidants and their role in influencing tissue regeneration via metabolic regulation are needed to determine whether reducing excess ROS in the stroma or stimulating the perturbation of cellular metabolism during wound healing may mitigate fibrotic processes to favor wound healing and scarless matrix deposition in KC.

Shared pathological mechanisms have been proposed for KC and Fuchs’ endothelial corneal dystrophy (FECD) involving elevated intracellular oxidative stress and reduced antioxidant responses, as previously reviewed [104,105]. FECD primarily affects older individuals and is associated with oxidative stress within the corneal endothelium that is thought to lead to the formation of aggregated extracellular matrix components in the form of guttae, the loss of corneal endothelial cells, and severe corneal edema. Increased lipid peroxidation has been detected in corneal tissue from patients with FECD showing ROS-mediated damage and progressive apoptosis of corneal endothelial cells [106]. These findings suggest that excess oxidative stress due to reduced antioxidant capacity or increased mitochondrial dysfunction within the corneal stroma and endothelial layer in the case of KC or FECD, respectively, may contribute to the loss of corneal transparency and significant defects in visual acuity.

## 4. Oxidative Stress in Cataract

The ocular lens is a transparent biconvex tissue suspended behind the iris by the lens zonules. Its highly organized architecture comprises two cell types: an anterior monolayer of cuboidal lens epithelial cells (LECs) and a mass of elongated lens fiber cells (LFCs), all encapsulated within a thickened basement membrane, known as the lens capsule [107]. The loss of lens transparency is known as cataract, which is the leading cause of blindness worldwide [108]. There are various types of cataract based on their anatomical location (nuclear, cortical, and subcapsular) and/or their etiology (age-related, diabetic, radiation, traumatic, and nutritional), the most common being age-related nuclear (ARN) cataract [109]. Currently, the only treatment for cataract is through surgical intervention, and while this initially results in a good visual prognosis, 35% of patients develop posterior capsular opacification (PCO), also known as secondary cataract, requiring further intervention to restore vision [110].

The proper functioning and longevity of the eye lens is achieved via several trade-offs. The removal of subcellular organelles, including ribosomes, from LFCs enables transparency [111] at the cost of protein turnover [112]. Encapsulation and devascularization of the lens help protect it from oxidative stress—oxygen’s partial pressure in the lens core is extraordinarily low [113]—though at the expense of potentially more efficient delivery of cellular reductants [114]. Similarly, an abundance of crystallin proteins (α, β, and γ in vertebrates) confers the lens with a high refractive index [115]; however, crystallins must maintain their folded and soluble state throughout life to ensure lens transparency [116]. As mature and metabolically quiescent LFCs lose their ability to synthesize and replace old proteins, crystallins accumulate post-translational modifications with age. Among these biochemical modifications are oxidation (including cysteine thiolation and disulfide formation), truncation, glycation, deamidation, and aspartate isomerization [117], as well as non-disulfide covalent crosslinking [118]. Individually or cumulatively, age-related modifications destabilize crystallins, leading to their partial unfolding and conversion to large protein aggregates that scatter light and cause the lens opacification characteristic of ARN cataract [117]. This age-related process is accelerated by environmental stresses such as exposure to ultraviolet radiation [119] and heavy metals [120]; the UV-light-induced oxidation of tryptophan residues in crystallins is highly correlative, with levels of intrinsic tryptophan fluorescence providing a measurable and reliable indicator of cataract severity [121].

The oxidation of methionine and cysteine residues is the chief modification in crystallins associated with ARN cataract [122,123]. The extensive disulfide linkages in crystallin aggregates from late-stage cataractous lenses have been recognized for decades [124,125,126], although other modifications such as deamidation are also prevalent [127,128]. There is growing evidence that oxidative and non-oxidative modifications, including deamidation [129,130], as well as amino acid mutations associated with inheritable, early-onset forms of cataract [131], act synergistically to predispose crystallins to aggregation and cataract formation. The molecular mechanisms underlying the oxidative misfolding and aggregation of crystallins have not been elucidated in detail. However, it is evident from the three-dimensional structures of the homologous βγ-crystallins that disulfide formation between pairs of natively buried, spatially distant cysteines (e.g., Cys32-Cys41 in γD-crystallin) [132] is detrimental, as such crosslinking necessitates major protein misfolding and consequent aggregation (e.g., via exposure of hydrophobic residues, dissociation of β strands) (reviewed in [133]).

Due to the long-lived nature of crystallin proteins, the eye lens has evolved a sophisticated antioxidant defense system with unusually high levels of cellular reductants such as GSH that are responsible for balancing the lens redox state [134,135]. An active GSH redox cycle exists in the lens epithelium and superficial lens cortex, whereby GSH detoxifies potentially harmful oxidants, such as dehydroascorbic acid and hydrogen peroxide [114,136]. Aside from the detoxification of hydrogen peroxide, GSH also functions as a key hydroxyl radical scavenger in LECs [137]. In the central lens nucleus, the levels of GSH are particularly low and even more so with increasing age. A developing diffusion barrier near the periphery of the lens nucleus [138], together with little to no regeneration of GSH, makes the lens nucleus especially vulnerable to oxidative stress and subsequent cataract formation. This susceptibility was elegantly illustrated in the LEGSKO mouse model, where de novo GSH synthesis in the lens is abolished via knockout of γ-glutamyl-cysteine ligase [139,140,141]. LEGSKO mice show increased oxidation of crystallin and non-crystallin proteins (at methionine and/or cysteine residues), leading to severe ARN cataract by 9 months [139,140,141]. Within the old and GSH-depleted regions of the lens, the cysteine-rich and abundant γ-crystallins may help buffer against oxidative stress by serving as redox sinks [142,143,144,145]. However, dynamic disulfide exchange between γ-crystallins succeeds only in delaying the oxidative protein aggregation cascade [143,146]. Eventually, with age, kinetically favorable yet relatively innocuous disulfides formed early in the lens are shuffled to more thermodynamically stable, yet deleterious, ones that have direr consequences for crystallin stability [133,143]. The development of small-molecule drugs capable of penetrating lens nuclei to restore the redox balance and/or stem aggregation remains the key challenge in the pharmacological prevention or treatment of ARN cataract [114,147,148,149].

Oxidative stress is also a key driver of the pathogenesis of fibrotic forms of cataract, including posterior subcapsular cataract (PSC) [150] and PCO. A key underlying mechanism of fibrotic cataracts involves epithelial–mesenchymal transition (EMT), whereby the once regular, cuboidal lens epithelial cells dramatically transform into spindle-shaped mesenchymal cells [151,152,153,154]. In PSC and PCO, the equatorial LECs undergo EMT and migrate posteriorly to form a fibrotic plaque that impairs vision. Transforming growth factor-beta 2 (TGFβ2) is a classic inducer of EMT in the lens, resulting in the enhanced migration, contraction, and secretion of extracellular matrix proteins [151,155,156]. The induction of oxidative stress through hydrogen peroxide-induced lens EMT is mediated by the enhanced activation of the TGFβ/Smad and Wnt/β-catenin pathways [157]. Two antioxidants, GSH and catalase, have been shown to potently block TGFβ-induced cataract in cultured rat lenses and lens epithelial explants [158]. The depletion of GSH in the lens leads to increased EMT markers, including vimentin, α-SMA, fibronectin, and collagen type I [159]. ROS-induced extracellular vesicles have been shown to enhance EMT of LECs, which can be suppressed by the addition of diphenyleneiodonium, an NADPH oxidase inhibitor [160]. The lens-specific knockout of glutamate-cysteine ligase catalytic subunit (Gclc), which encodes the rate-limiting enzyme in GSH synthesis, resulted in elevated oxidative stress, inflammation, and the robust upregulation of cytokines [161]. The professional ROS producer, NADPH oxidase 4 (Nox4), has been shown to be upregulated in response to the TGFβ2 stimulation of rat lens epithelial explants, leading to EMT [162]. The blockade of Nox4 using a specific NADPH oxidase inhibitor reduced the TGFβ-induced upregulation of the gene expression of α-SMA, collagen 1a, and fibronectin [163], further emphasizing the key role of oxidative stress in driving lens EMT in the pathogenesis of fibrotic cataract.

## 5. Retina

The retina acts as the “sensor” of the eye, where the light is focused to interpret visual information. It is a highly organized tissue composed of nine histological layers comprising 50 different cell types, all resting on a pigmented epithelium. The retinal layers consist of photoreceptors (PRs), the external limiting membrane (ELM), the outer nuclear layer (ONL), the outer plexiform layer (OPL), the inner nuclear layer (INL), the inner plexiform layer (IPL), the ganglion cell layer (GCL), the nerve fiber layer (NFL), and the internal limiting membrane (ILM) [164,165].

The PRs in the outermost layer of the retina are responsible for transforming light energy into nerve impulses. There are approximately 100 million PRs distributed throughout the human retina, making them one of the most abundant types of neurons in the body, highlighting the herculean nature of image capture and processing [166,167]. The transformation of light energy by the photoreceptors is dependent on the alteration of visual pigments contained within the rods and cones [168]. These pigments consist of a Vitamin A-derived aldehyde, known as retinal, bound to large protein moieties, called opsins, which differ depending on the type of photoreceptor [164]. Light isomerizes retinal 11-*cis* to all-*trans*, releasing retinal from the protein moiety, a chemical sequence that promotes the transient excitation of the photoreceptor that propagates along its axon to stimulate second-order neurons, notably bipolar and amacrine cells [169]. These cells synapse onto retinal ganglion cells (RGCs) in the INL, whose axons coalesce to form the NFL and optic nerve, terminating at the lateral geniculate nucleus, which is connected by a second set of neurons to the visual cortex of the brain [170,171]. The integrity of the PR layer is heavily influenced by the retinal pigment epithelium (RPE), which consists of cells with sheet-like microvilli that interact with the outer PR segments extending from the outer retinal surface [172,173]. The RPE is essential in maintaining retinal homeostasis by facilitating the directional transport of nutrients and the clearance of photooxidized outer segment membranes [173]. Additionally, the RPE physically interacts with the outer retinal blood supply, known as the choroid, forming a structure known as the outer blood–retinal barrier (BRB). The choroid consists of five histological layers: Bruch’s membrane, the choriocapillaris, Sattler’s layer, Haller’s layer, and the suprachoroid [174].

The retina contains three major types of glial cells: microglia and two types of macroglia, astrocytes and Müller cells. Microglia are the primary innate immune cells of the retina. Suggested to be a heterogeneous population, they are in the GCL, in both plexiform layers, and around the vasculature as different subclasses [175,176]. Microglia constantly engage in the surveillance of surrounding neural tissue and contribute to the host defense against microorganisms, initiating inflammatory responses and promoting tissue repair in close association with macroglia and blood-borne immune cells [177,178]. Other roles for microglia include supporting vascular growth, the formation of neural connections, and neuronal apoptosis [179,180,181]. Given this association, microglial activation is thought to contribute to the progression of retinal degenerative diseases [182]. Astrocytes are primarily localized to the NFL and GCL and also exist as a heterogeneous population of three morphological subclasses interacting with nerve fibers, blood vessels, or the space between [183,184,185]. However, they are most well known for their role in supporting the retinal vasculature by ensheathing blood vessels, secreting trophic factors such as vascular endothelial growth factor (VEGF), and maintaining the integrity of the blood–retinal barrier [186,187].

Müller cells, the most common type of glial cell in the retina, extend radially across the entire retina to support cells in both the ILM and OLM [188]. Due to this orientation, Müller glia contact virtually every other retinal cell type, structurally minimize intraretinal light scattering, and help light focus onto the photoreceptors [189]. Müller cells provide critical homeostatic and trophic support to both the retinal vasculature and neuronal layers [190]. Among their many roles, Müller cells recycle neurotransmitters to prevent excitotoxicity, provide the spatial buffering of ions, reabsorb fluid to prevent edema, participate in the retinoid (visual) cycle, and regulate nutrient levels. In addition, Müller cells release a variety of neurotrophic and angiogenic growth factors and cytokines, as well as critical antioxidants such as GSH [191]. Müller cells are uniquely sensitive to metabolic perturbations and can become dysfunctional as they try to counter-regulate them [192,193]. This phenomenon is referred to as gliosis and is characterized by morphological and functional changes in the Müller glia. These changes manifest, in part, as alterations in gene and protein expression that contribute to retinal inflammation, microvascular defects, and neuronal dysfunction [192]. Prolonged or extensive damage can also induce metabolic memory, causing Müller cells to remain in a state of reactive gliosis even after the deleterious stimulus has dissipated.

### 5.1. Oxidative Stress and Retinal Vasculature

The retina is one of the most highly metabolically active tissues in the body and requires significant levels of oxygen and nutrients to maintain normal visual function. They are supplied by two key vascular networks: the central retinal artery system and the choriocapillaris [194]. Vessels extending from the central retinal arteries supply the inner half of the retina, branching into superficial, intermediate, and deep capillary layers after entering the eye via the center of the optic nerve. The choriocapillaris, directly adjacent to Bruch’s membrane, perfuses the outer retina and nourishes the RPE and photoreceptors [195]. Multiple cell types have been implicated in the development and maintenance of the retinal vasculature. RGCs are crucial contributors to maintaining the integrity of these vessels through the secretion of angiogenic factors and interact closely with them as the GCL is perfused by the superficial and intermediate vascular plexuses [196,197,198]. Pericytes are mural cells associated with the microcirculation that wrap around the endothelial cells that line systemic capillaries. Located in the basement membrane, they interact with the endothelium through both physical contacts that penetrate the basal lamina and via amacrine signaling. In the retina, they help support the vasculature, blood flow, and the BRB and respond to proangiogenic factors secreted by other cell types, such as RGCs. Combined with the metabolic and homeostatic support provided by glial cells, these cells coalesce to form the neurovascular unit to regulate retinal blood flow through vasodilation and vasoconstriction [199,200,201,202].

Given the high metabolic demand of the retina, its energy requirements are primarily met through oxidative and glycolytic metabolism with the input of additional nutrients by the retinal vasculature [203,204]. This can result in ROS/RNS production, which are tightly controlled by retinal glia and play important roles as secondary messengers in endothelial features [205,206]. Yet, under pathological conditions, these reactive species can accumulate, impacting the neurovascular unit and ultimately causing vascular disease [199,207]. For example, ROS accumulation causes an imbalance in nitric oxide metabolism, which impairs the response of vascular endothelial and smooth muscle cells to inflammation and changes in blood flow. Other consequences include increases in vascular permeability and retinal endothelial cell apoptosis [208,209]. These can occur as a result of a variety of pathological conditions and contribute to their overall progression while further exacerbating ROS production. The following sections discuss some of the more severe retinal diseases heavily associated with oxidative stress.

### 5.2. Oxidative Stress during Age-Related Macular Degeneration

Age-related macular degeneration (AMD), a retinal neurodegenerative disorder, is a common cause of irreversible central visual impairment in the aging population [210,211]. This ocular disease causes damage to the macular region of the retina, an oval-shaped, yellow area located in the central retina that facilitates high visual acuity and color vision due to the presence of the highest density of cone photoreceptors [212]. AMD is associated with a multifactorial etiology, with factors including increased age, female sex, obesity, genetics, a high-fat diet, and smoking [213]. There are two major forms of AMD: (i) dry AMD, which accounts for 90% of diagnosed AMD cases and is characterized by the formation of drusen between Bruch’s membrane and the retinal pigment epithelium (RPE), as well as the RPE and photoreceptor degeneration [214], and (ii) wet AMD, which is characterized by choroidal neovascularization (CNV) and involves the formation of new and leaky blood vessels driven by VEGF secretion, leading to macular edema, hemorrhage, and fibrous tissue proliferation [215,216].

**Figure 2:**
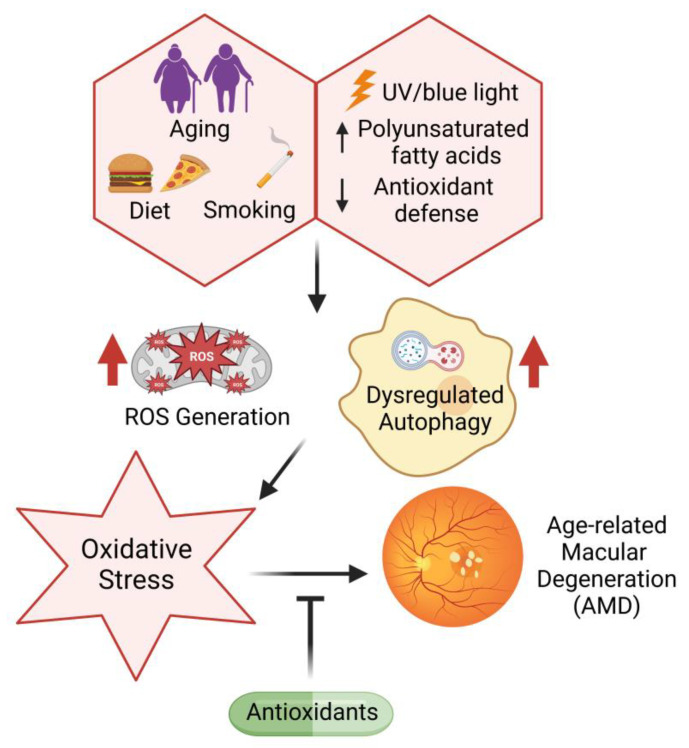
Factors driving oxidative damage during age-related macular degeneration (AMD) and the need for antioxidant defense systems to combat the pathophysiology of the disease.

The retinal microenvironment is prone to oxidative damage due to high oxygen consumption [217], exposure to visible light (400–700 nm), and the presence of high levels of polyunsaturated fatty acids and photosensitive molecules, including, lipofuscin and rhodopsin. With age, the decreased antioxidant capacity further accentuates the oxidative components, creating a harsh retinal microenvironment [218]. These age-related oxidative changes are central to AMD pathogenesis [219] (Figure 2), with increased ROS causing damage to cellular lipids, proteins, and DNA and impairing retinal function [220]. Previous studies have documented the presence of systemic oxidative stress in patients with AMD in comparison to non-AMD cohorts, indicated by the upregulation of MDA, 8-OHdG, and protein carbonyls [221]. Additionally, reduced levels of nitric oxide synthase (NOS) isoforms—neuronal (nNOS), inducible (iNOS), and endothelial (eNOS)—play an important role in AMD pathogenesis [222]. On the other hand, at high concentrations, NO is known to react with superoxide anions (O_2_^·−^) to generate peroxynitrite (ONNO^−^), which has the potential to accumulate protein aggregates between the RPE and photoreceptors, leading to their degeneration [223,224].

It has been documented that RPE cells derived from AMD patients lose their capacity to upregulate SOD expression upon oxidative stress exposure, thus leading to increased ROS accumulation compared to non-AMD RPE cells [225]. Moreover, mice deficient in SOD1 and SOD2 exhibit elevated levels of ROS, along with the development of specific features of AMD pathology, such as drusen formation, Bruch’s membrane thickening, and choroidal neovascularization [226,227]. Another antioxidant that plays an important role in the antioxidant response and retinal detoxification is nuclear factor erythroid 2-related factor 2 (NFR2). Downregulation of the NFR2 gene has been shown to increase retinal degeneration and lead to the development of an AMD-like phenotype with dysregulated autophagy, thus indicating a link between inflammation and oxidative stress [228]. A known Nrf2 activator, dimethyl fumarate, has been shown to suppress inflammation and metabolic dysfunction of the RPE [229] as well as blue-light-induced oxidative damage via the Nrf2 pathway [230]. Further, the accumulation of advanced glycation end products (AGEs) has been observed in the macular drusen of AMD patients, which are linked to enhanced inflammation, oxidative stress and vascular dysfunction [231,232]. One of the potential biomarkers in determining AMD susceptibility is carboxyethylpyrrole (CEP). Proteomic analyses of AMD patients have indicated significantly elevated levels of carboxyethylpyrrole protein adducts in their drusen [233]. CEPs are generated by the oxidation of the polyunsaturated fatty acid docosahexaenoic acid (DHA) in the outer segments of photoreceptors, making it a robust oxidative stress marker for AMD patients [234].

The retinal absorption of ultraviolet rays causes photochemical damage to mitochondria in the RPE, leading to increased ROS generation [235,236]. Specifically, blue light has been associated with significant photooxidative stress and ROS generation [237,238]. Light-induced damage is dependent on various chromophores, among which flavins and porphyrins have been identified as possible chromophores responsible for mitochondrial damage induced by blue light [239,240]. Moreover, the macular carotenoid lutein has antioxidant properties but also prooxidant properties at high concentrations [241]. Blue-light-mediated oxidative stress is proposed to occur in the outer photoreceptor segments through the NOX family of enzymes [242]. NOX2 and NOX4 increase ROS levels in photoreceptors irradiated with blue light, which has been shown to decrease with apocynin, a NOX inhibitor [242].

Another important risk factor for AMD is cigarette smoking. Studies have documented a 2–3-fold elevated risk of developing AMD in smokers compared to non-smokers [243]. Cigarette smoking has the ability to generate ROS and increase oxidative stress by elevating serum lipid peroxidation [244] and reducing the levels of antioxidants [245]. Additionally, it increases the levels of proinflammatory cytokines, including C-reactive pprotein (CRP) [246], which is associated with an increased risk of AMD. Notably, in vascular smooth muscle cells, CRP is known to exhibit prooxidative effects, triggering ROS accumulation and subsequent apoptosis [247] and promoting the adhesion of monocytes to endothelial cells through NOX-mediated oxidative stress [248]. Recently, studies have documented elevated levels of interleukin 1 beta (IL-1β), TNFα, and iNOS in mouse retinas following exposure to electronic cigarette vapor [249]. Furthermore, smoking is correlated with genetic susceptibility to AMD. For instance, the complement factor H Y402H polymorphism along with smoking has been associated with AMD [250].

In addition to environmental stressors and exogenous ROS, intracellular defects, such as dysregulated autophagy, have also been implicated in AMD progression. The impairment of the autophagic pathway leads to the decreased clearance of damaged organelles and proteins that can exacerbate oxidative stress and contribute to AMD pathogenesis [251]. In turn, the promotion of autophagy through the downregulation of hyperactive mTORC1 signaling by upregulating calcium and integrin binding protein 2 (CIB2) and the activation of the proliferator-activated receptor gamma coactivator 1 (PGC-1) [252] and AMPK-mTOR [253] signaling pathways have shown significant potential as novel therapies for dry and wet AMD [254,255]. Mitochondria play an important role in ROS generation since the ROS produced in the ETC can cause damage to cellular components. Previous studies have reported elevated levels of mtDNA and decreased DNA repair efficacy with the progression of AMD [255]. The downregulation of two key regulators of mitochondrial biogenesis and antioxidant production (nuclear factor erythroid 2-related factor 2 (NFE2L2) and PGC-1α) cause a disturbance in mitochondrial autophagy, known as mitophagy [256]. Previous reports have documented a decrease in mitophagy along with the accumulation of damaged mtDNA during AMD [257,258]. Along with this, the accumulation of iron in the retina has been observed in AMD. Excess iron is associated with ROS generation through the Fenton reaction [259]. In hereditary disorders such as hemochromatosis, which involves systemic iron overload, iron accumulation has been observed in the retina in spite of an intact blood–retinal barrier (BRB), resulting in clinical signs of AMD [260,261]. Iron-mediated oxidative stress has been reported in a sodium iodate-induced dry AMD model, indicating the significant upregulation of ferritin and impaired cathepsin D synthesis and maturation, resulting in iron-loaded ferritin accumulation [262].

At present, anti-VEGF therapy is approved for wet AMD. Various anti-VEGF agents, including aflibercept [263,264], bevacizumab, ranibizumab [265,266,267], and more recently, brolucizumab [268,269], have been used for the treatment of wet AMD. However, therapeutics for dry AMD remain limited. Numerous agents have been evaluated in the past for dry AMD treatment, including anti-inflammatory agents [270], immunomodulators, and antineoplastic agents [271]. Recently, a randomized phase II trial documented the efficacy of a complement 3 inhibitor, pegcetacoplan, in significantly reducing the progression of geographic atrophy in AMD patients [272].

Studies have supported the use of antioxidants in oxidative-stress-induced AMD models. Antioxidants such as zinc, resveratrol, and carotenoids have been proposed for dry AMD treatment [273,274]. Based on the Age-Related Eye Disease Study (AREDS), oral antioxidants are effective supplements for slowing AMD progression. Daily supplementation with select antioxidants, such as Vitamin C, Vitamin E, and beta-carotene along with zinc, was found to reduce the risk of AMD [275]. However, the follow-up clinical study, called the Age-Related Eye Disease Study 2 (AREDS2), testing the effects of further supplementation with omega-3-fatty acids DHA and EPA, along with carotenoids, lutein, and zeaxanthin, indicated no further reduction in AMD progression [276]. Supplementation with compounds such as Vitamin E, which has the ability to inhibit lipid peroxidation [277], has also shown protective properties in the RPE. Alpha-tocopherol transfer protein (α-TTP) has been associated with the maintenance of serum Vitamin E levels. The deletion of the α-TTP gene has been linked with retinal degeneration and the loss of photoreceptors. On the other hand, supplementation with Vitamin E was shown to suppress lipid peroxidation in a mouse model of neuronal degeneration [278]. While these collective findings suggest that supplementation with select antioxidants may delay AMD progression, further clinical trials are needed to establish therapeutic efficacy in larger, diverse populations and to develop preventative interventions to reduce retinal degeneration with age.

### 5.3. Oxidative Stress and Proliferative Vitreoretinopathy

Proliferative vitreoretinopathy (PVR) is a complication of rhegmatogenous retinal detachment surgery or severe ocular trauma, including intraocular foreign bodies, perforation, penetrating injury, contusion, or globe rupture [279]. Clinically, PVR is characterized by the formation of contractile fibrotic membranes on the epiretinal, intraretinal, and/or subretinal surfaces [280]. The rate of PVR is approximately 5–10% of patients undergoing retinal detachment surgery [281], and of these patients, less than 25% will achieve a visual acuity of 20/200 or better [282]. The only treatment for PVR is through the surgical excision of the fibrotic membrane [280]. The retinal cells identified in patient PVR membrane specimens predominantly comprise RPE but also glial cells (primarily Müller cells) and inflammatory cells (lymphocytes and macrophages). Interactions between these cells with a myriad of growth factors and cytokines derived from vitreous contact and breach of the blood–retinal barrier trigger a cascade of downstream cellular processes, including EMT, chemotaxis, proliferation, excessive extracellular matrix deposition, and cellular migration, leading to the formation of contractile fibrotic membranes and the overt loss of the retinal architecture [280].

Alterations in redox homeostasis have been implicated in the pathogenesis of PVR. Verdejo et al. (1999) examined the vitreous of patients undergoing vitrectomy for PVR, proliferative diabetic retinopathy, rhegmatogenous retinal detachment, macular hole, or epiretinal membrane [283]. Increased free radial formation and reduced antioxidant activity (SOD and catalase levels) were observed in the vitreous of PVR patients [283]. An increase in oxidative stress, proportional to disease severity, has been found in the vitreous of patients undergoing surgery for rhegmatogenous retinal detachment compared to macular hole [284,285]. Intriguingly, it has been observed that the retina may launch a protective response to injury by increasing its antioxidant defense system. Increased levels of the radical scavenger α1-microglobulin were found in patients with rhegmatogenous retinal detachment compared to macular hole [284]. Further support for launching a protective antioxidant response was shown by Pietras-Baczewska et al. (2021), where increases in two antioxidative enzymes, SOD and glutathione reductase, were detected in the vitreous of patients with PVR compared to patients with macular hole or epiretinal membrane [286]. Regardless of the discrepancies in the precise changes in oxidative stress and antioxidant levels, it is clear that an imbalance of ROS and antioxidant defenses occurs during PVR. This may trigger subsequent damage, including lipid peroxidation, cellular degeneration, and the release of diverse regulatory molecules and cytokines that drive the further progression of the disease. Reduced GSH concentrations in vitreous and blood samples of PVR patients have been detected [287]. Oxidative tissue damage through lipid peroxidation, as measured by thiobarbituric acid-reactive substances, has been identified in epiretinal membranes isolated from patients with PVR [288].

EMT of the RPE is a predominant process driving PVR pathogenesis [289]. During EMT, the regular and polarized RPE cells transform into motile, matrix-producing mesenchymal cells that secrete and deposit excessive extracellular matrix [290,291]. These transdifferentiated myofibroblasts migrate along the subretinal plane and enter the epiretinal plane by migrating through retinal breaks [280]. TGFβ is the primary growth factor involved in the induction of EMT of the RPE and is present at high concentrations in vitreous isolated from PVR patients [292]. Oxidative stress was found to enhance TGFβ-induced EMT through the upregulation of the inflammatory cytokine chemokine ligand 1 (CXCL1) in the ARPE-19 cell line [293]. In this study, hydrogen peroxide induced the upregulation of CXCL1 which, in turn, upregulated mesenchymal markers such as α-SMA and fibronectin in the RPE [293], highlighting a synergistic effect with inflammation, oxidative stress, and EMT. While RPE cells physiologically express NOX1, NOX2, and NOX4, dysregulation of NOX4 signaling has been shown to augment EMT [294,295,296]. VAS2870, a novel NAPDH oxidase inhibitor, blocked the TGFβ-induced EMT of the RPE [297]. The administration of an antioxidant, αB crystallin peptide, significantly reduced vimentin, fibronectin, and CTGF levels in a dispase-induced PVR mouse model and, in doing so, improved the mitochondrial function of the RPE [298]. Other antioxidants, such as quercetin [299], ZLN005 [300], and artesunate [301], have shown efficacy in blocking the TGFβ-induced EMT of the RPE, further highlighting a key role for oxidative stress in PVR pathogenesis.

### 5.4. Oxidative Stress and Diabetic Retinopathy

Diabetic retinopathy (DR) is the leading cause of acquired blindness in working-age adults in Western countries, with approximately 90% of diabetic individuals experiencing vision-threatening complications within 25 years of their initial diagnosis [302]. In the clinic, DR is primarily characterized by vascular abnormalities that can be detected by current diagnostic techniques. These include microcirculatory complications that affect the capillaries in the eye, such as microaneurysms, hemorrhaging, and angiogenesis, which are all associated with the more severe, late-stage aspects of DR that can lead to retinal detachment and blindness [303].

In addition to vascular abnormalities, the neurosensory retina is altered in diabetes. The neurosensory retina generates vision but is transparent and largely undetectable by standard clinical examination. However, most retinal neurons and glial cells are altered prior to the development of microvascular lesions and are progressively impaired with worsening retinopathy. These alterations include biochemical defects, such as impaired control of glutamate metabolism (the major neurotransmitter) [304], as well as the loss of synaptic activity and dendrites [305], apoptosis of neurons primarily in the ganglion cell and inner nuclear layers [306,307], and the activation of microglial cells that may protect the inner retina from injury and contribute to the inflammatory response [308].

With improvements in understanding the pathogenesis of diabetes over the past several decades, it has been well accepted that most complications associated with diabetes are linked to the severity of hyperglycemia. Two of the most comprehensive studies exploring this relationship, The Diabetes Complications and Control Trial (DCCT) and the UK Prospective Diabetes Study (UKPDS), report that hyperglycemia is the dominant contributor to the manifestation of DR in both type 1 and type 2 diabetes [309,310]. Both reports also indicate a significant reduction in DR development with intensive glycemic control. Four key pathways have been implicated in the effects of hyperglycemia-induced tissue damage: the polyol pathway, AGE pathway, hexosamine biosynthetic pathway, and protein kinase C pathway.

A unifying theory for the pathobiology of diabetic complications suggests that the activation of each of these pathways is linked to the excessive production of ROS [311]. While acute bursts of ROS are important in normal physiological signaling through their role as secondary messengers, prolonged exposure can result in damage to macromolecules [312]. This includes proteins, lipids, and nucleic acids, whose modification by ROS can impede normal physiological function [313]. Thus, retinal cells must maintain the tight regulation of ROS by balancing their production and clearance [314]. Sources of ROS within the retina include NADP(H) oxidases, xanthine oxidases, and retinal excitation by light [315,316,317,318]. Yet, the dominant source of retinal ROS is the mitochondrial ETC [319,320]. Photoreceptors and the RPE are two retinal cell types that have been found to contain a high mitochondrial density. Previous work suggests that hyperglycemia increases ΔΨ_m_, which leads to excess superoxide production through enhanced glycolytic flux and a surplus of electron carriers generated by the TCA cycle [321,322,323,324]. The carriers enter through ETC complexes I and II, resulting in a ΔΨ_m_ that overloads complex III and generates superoxide [325]. Additional evidence substantiates this by describing a multicomponent feedback loop that continues to drive this harmful production of ROS [326,327]. Non-enzymatic antioxidants such as β-carotene, Vitamin C, and Vitamin E are all negatively impacted by hyperglycemia [328]. Enzymatic factors, which include proteins such as SOD, catalase, and enzymes involved in the synthesis of GSH, are also negatively influenced by hyperglycemia [329,330,331,332].

Increases in reactive nitrogen species (RNS), including the nitroxyl anion, the nitrosonium cation, and peroxynitrite, are also coincident with elevated ROS. It has been reported that the increased activation and expression of eNOS is commonly seen in models of type 1 and type 2 diabetes and can result in elevated levels of NOS that can ultimately cause neurological and vascular complications [333,334,335,336]. While nitric oxide is also important in cellular signaling, it can react with O_2_^2−^ to form a peroxynitrite radical, which is thought to have cytotoxic effects under hyperglycemic conditions [337,338,339].

**Figure 3:**
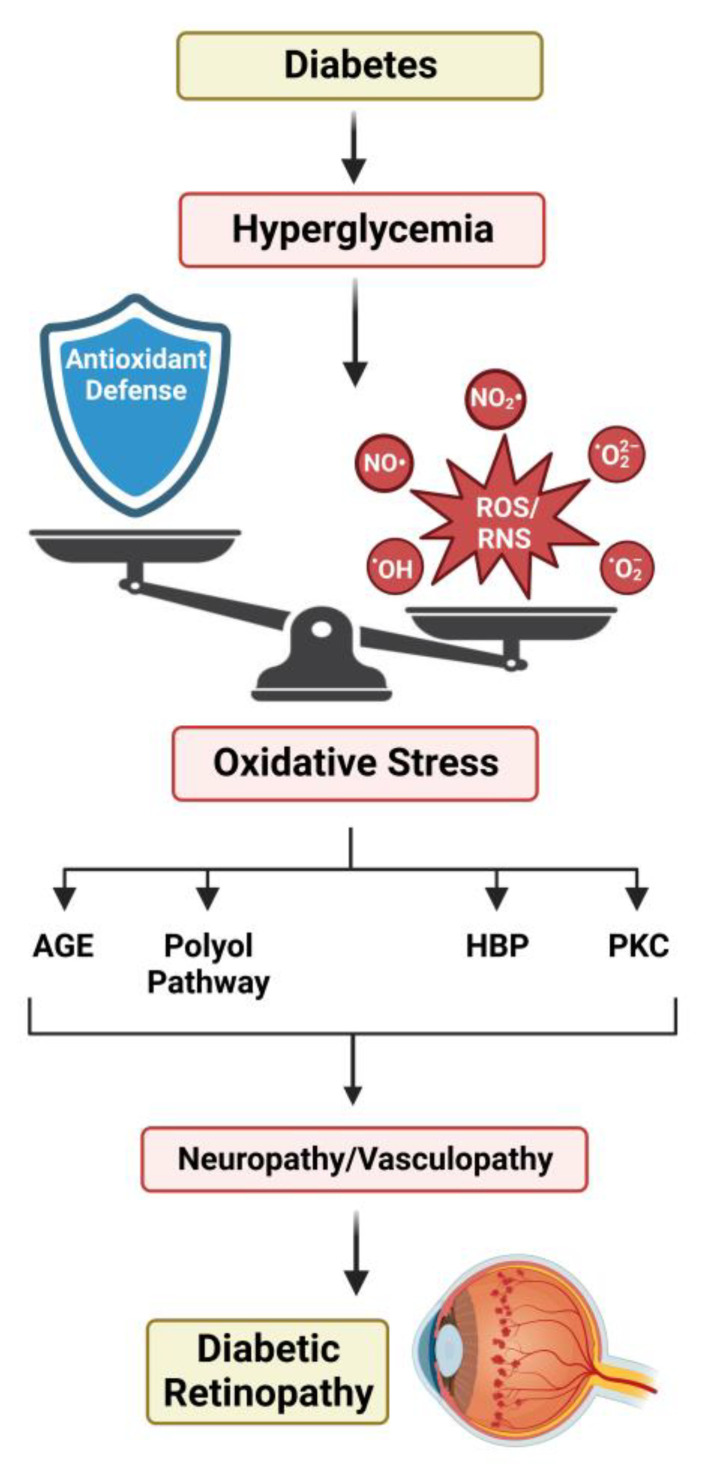
Diabetes-induced hyperglycemia disrupts the redox balance, leading to oxidative stress in the retina. Under normal physiological conditions, there is a balance in the production of reactive oxygen species (ROS)/reactive nitrogen species (RNS) and the antioxidant defense system. Diabetes-induced hyperglycemia promotes ROS/RNS generation while also suppressing the retinal antioxidant response, creating the imbalance known as oxidative stress. The AGE pathway, the polyol pathway, the hexosamine biosynthetic pathway (HBP), and the protein kinase C pathway are all sensitive to this disruption and play an important role in the downstream effects of hyperglycemia-induced retinal damage.

In addition to ROS and RNS, studies have demonstrated that diabetes-induced hyperglycemia causes a failure to upregulate factors associated with the antioxidant response [340,341,342,343]. Notably, patients diagnosed with type 1 and type 2 diabetes have been shown to exhibit a significant reduction in antioxidants [344,345,346]. This exacerbates the accumulation of harmful reactive species, creating the imbalance known as oxidative stress, contributing to the hyperglycemia-induced tissue damage that drives DR pathogenesis (Figure 3).

Hyperglycemia compromises the antioxidant response [347,348,349], owing, in part, to enhanced degradation due to the dysregulated diabetic metabolic microenvironment [343]. The Nrf2 gene battery consists of more than 200 genes associated with not only the redox balance but also processes such as inflammation and proteostasis [350]. Thus, its augmentation has been of great interest in developing therapies to combat DR.

The retina contains several different cell types that exhibit high metabolic activity through the consumption of large quantities of glucose and oxygen [351]. It also possesses some of the highest concentrations of PUFAs among all the tissues in the body, making it sensitive to lipid peroxidation [352]. As a result, any changes in the redox balance due to diabetes-induced hyperglycemia are likely to alter retinal structure and function. Previous reports have demonstrated a clinical correlation between free radical formation and the development of DR. This has also been supported by several in vivo studies that report the accumulation of ROS [315,321,327,353,354,355] and RNS [356,357,358,359] in retinal tissues of diabetic rodents. The exposure of endothelial cells, pericytes, neurons, and Müller cells to hyperglycemic conditions has been shown to result in the accumulation of these free radicals and can progress to retinal cell apoptosis [327,329,360,361,362,363]. Müller cells, astrocytes, and photoreceptors have also been shown to be affected early in DR pathogenesis, resulting in elevated ROS/RNS levels that cause further damage to these retinal cells [343,364,365,366].

The suppression of the antioxidant defense has been reported in multiple DR models [343,367]. Pericytes isolated from the postmortem retinas of diabetic patients exhibited the downregulation of mRNAs encoding glutathione reductase and SOD upon exposure to hyperglycemic culture conditions when compared to those from non-diabetic patients [368]. In both postmortem samples and preclinical models from diabetic patients, retinal Nrf2 DNA-binding activity was found to be reduced [26,343,369]. In rodents, streptozotocin (STZ)-induced diabetes suppressed retinal GSH levels and increased lipid peroxidation [370,371]. In vitro studies exposing bovine pericytes to AGEs have also documented a decrease in intracellular catalase and SOD activities [372].

Antioxidant supplementation has become an avenue of interest for combatting DR pathogenesis by addressing earlier deficits in the neurosensory retina that are not targeted by current therapeutics, while also hindering progression to later stages of the disease. In diabetic rats, the administration of Vitamins C and E reduced the appearance of acellular capillaries and pericyte ghosts and halted DR progression in alloxan-induced diabetic rats [329]. In vitro treatment with α-lipoic acid and N-acetyl cysteine, the precursor of the amino acid cysteine required for GSH synthesis, inhibited oxidative-stress-induced damage in retinal endothelial cells, pericytes, and neuronal precursor cells [327,373]. The administration of α-lipoic acid also prevented retinal capillary cell death and the development of microvascular defects in diabetic rats [329]. Dietary N-acetyl cysteine supplementation attenuated the development of vascular pathology in the retina of STZ-treated diabetic rats and has been shown to prevent retinal cell death and contrast sensitivity deficits in STZ-treated diabetic mice [327]. Unfortunately, data obtained from multiple clinical trials showed no protective effect of β-carotene or Vitamin C and E supplementation in DR patients [374,375,376]. However, this does not discount the potential benefit of modulating ROS-generating or antioxidant defense pathways as therapeutic options in DR.

### 5.5. Oxidative Stress Mechanisms in Glaucoma

Glaucoma is the second leading cause of blindness, with estimates of 80 million glaucoma patients expected to be diagnosed by 2040 [377]. Glaucoma patients are characterized by a progressive loss of RGCs and their axons and subsequent visual field loss. Elevated intraocular pressure (IOP) is a well-known risk factor for glaucoma and is a diagnostic clinical sign of primary open-angle glaucoma (POAG) [378]. Elevated IOP and dysregulated blood flow in the retina may cause initial damage to the optic nerve. Current glaucoma therapies merely slow disease progression, with no treatment available for reversing glaucomatous neurodegeneration. Thus, exploration of the underlying mechanisms is critical for designing novel treatments for glaucoma. Glaucoma is a multifactorial retinal degenerative disease that manifests as different subtypes, such as POAG and primary angle closure glaucoma (PACG). POAG is the more common form of glaucoma compared to PACG [377]. In normal-tension glaucoma (NTG), patients present with glaucomatous optic neuropathy but exhibit IOP in the normal range, that is, between 11 and 21 mmHg. NTG is more common in Asian populations (52–92%) [379] and presents in 30–40% of Caucasian patients with POAG [380].

The mechanisms of the progressive degeneration of RGCs and their axons in glaucoma have been attributed to various factors, such as the elevation of IOP, the activation of immune cells, altered trabecular meshwork (TM) cells, and dysregulated gut microbiota. Oxidative stress is one of the candidates leading to POAG and RGC degeneration. The antioxidant capacity in patients with POAG is reduced by over 60%, suggesting that these patients might be more susceptible to oxidative stress of the TM and retinal tissues [381,382].

The retina is the most energy- and oxygen-demanding tissue in the body and exhibits intense mitochondrial activity [383]. RGCs consume 5-fold more ATP than photoreceptors in the dark [384]. The imbalance of ROS and antioxidant enzymes are critical to the health of retinal neurons. Excess and unstable ROS may react with lipid membranes, proteins, and nucleic acids, leading to RGC degeneration and apoptosis [385,386]. Elevated IOP may affect the drainage facility of TM cells and reduce blood flow [387], imposing stress on retinal structures, especially the anterior segment of the optic nerve. This interferes with axonal transport, leading to RGC degeneration. Oxidative stress is considered one of the major drivers of damage to TM cells in glaucoma [388]. TM cells could be damaged by elevated ROS levels and lead to alterations in the drainage facility of the TM [389].

In addition to ROS production, oxidative stress could affect DNA modifications [390] through alkylation, deamidation, and oxidation, eventually leading to cell apoptosis [12,391]. These DNA modifications can be repaired by base excision repair mechanisms [390]. An association between base excision repair gene polymorphisms and a higher risk of POAG in Polish populations has been reported [392]. Clinical studies have shown the upregulation of a DNA oxidation marker, 8-OHdG, in the blood, aqueous humor, and TM from POAG patients [393]. Changes in 8-OHdG in the TM have been correlated with visual field defects [393]. As further support for the impact of oxidative stress on DNA in glaucoma, a negative correlation between base excision repair gene expression (PARP1 and OGG1) and the 8-OHdG level has been detected in the plasma of glaucoma patients [394]. The chronic exposure of TM cells to oxidative stress leads to reduced cell viability and increased cell senescence [395] via the upregulation of cyclin-dependent kinase inhibitors p16 and p21 [396]. Senescent cells are the active source of proinflammatory cytokines, chemokines, and extracellular-matrix-degrading proteins that may promote a toxic environment for RGCs [397,398].

The response to oxidative stress is mainly regulated by the mitochondria of RGCs and their axons [399]. In a study using the DBA/2J mouse model of congenital glaucoma, mitochondrial dysfunction was found to be the key driver of early neuronal dysfunction before RGC degeneration [400]. NAD is a highly conserved coenzyme central to metabolism and serves as an important redox cofactor, providing electrons to complex I of the ETC for ATP production. Nicotinamide treatment was shown to protect against RGC loss in DBA/2J mice [401], which is further supported by observations of NAD deficiency in POAG patients [402]. Mitochondrial dysfunction releases ATP and ROS, activating the NLRP3 inflammasome and releasing potent inflammatory signals [403]. Excessive ROS can be neutralized by antioxidant defense mechanisms or enzymes, including SOD, glutathione peroxidase, catalase, and glutathione reductase [404]. Accumulating evidence supports redox dysregulation in glaucoma patients, revealing an imbalance in ROS and antioxidant levels. Intriguingly, levels of individual antioxidant enzymes do not always show reduced expression in glaucoma patients. For example, the expression of SOD1 was downregulated in blood samples from POAG patients, but SOD2 expression was upregulated [405]. The knockout of antioxidant-defense-system-related genes (SOD1, SOD2, NRF2) in animal models typically results in progressive neuro- and retinal degeneration, with loss of RGCs, decreases in ERG waves, and increases in cleaved-caspase-3-positive apoptotic cells in the retina and varying degrees of disease progression with age [227,228,406]. However, none of these models investigated the effect of elevated IOP, so the conclusions derived from these studies are for general retinal neurodegeneration.

NOX enzymes are also key contributors to oxidative stress. Excessive oxidative stress may generate free radicals, such as the superoxide anion, hydroxyl radical, and nitric oxide. NOX, xanthine oxidase, uncoupled NO synthase, and defects in the ETC lead to excessive ROS generation [407,408], especially the latter one [383,384,409]. The catalytic unit of NOX has seven isoforms, NOX1–5 and DUOX1–2. NOX expression has been implicated in the pathogenesis of various animal models of glaucoma [410]. NOX1 and NOX2 generate superoxide, and NOX4 generates hydrogen peroxide [411]. Disruption of the NOX system in animal models, however, primarily leads to photoreceptor degeneration [412], whereas the primary feature of POAG is RGC loss.

RNS such as uncontrolled nitric oxide production (NO, a well-known vasodilator) can induce oxidative stress, leading to apoptosis [413]. The upregulation of iNOS and the downregulation of calcium/calmodulin-dependent NOS expression were correlated with visual defects in POAG patients [414]. Endothelin-1 (ET-1), a well-known vasoconstrictor, has been shown to increase in POAG patients [415,416,417]. Dysregulation of vasodilation and vasoconstriction could affect the RGC metabolic supply and increase oxidative stress, leading to RGC degeneration [418]. Clinical studies have shown that total biological or total reactive antioxidant levels are generally reduced in blood samples collected from glaucoma patients, correlating with glaucoma severity [419,420,421,422]. Furthermore, increased MDA levels, a well-known secondary product of lipid peroxidation [423], in the blood and aqueous humor [424], and reduced antioxidant levels [424,425] have been detected in glaucoma patients.

Microglial activation is an early pathophysiological change that has been shown to precede RGC loss in both glaucoma patients and the glaucomatous DBA/2J mouse model [426]. Transcriptome studies in microglia in the optic nerve head indicate significant changes in metabolic, phagocytotic, inflammatory, and sensome-related genes [427]. The upregulation of the major proinflammatory cytokine TNF-α is well-known in glaucoma [428,429,430,431]. Both TNF-α and heat shock proteins (released from damaged, stressed, or dead cells) upregulate NOX activities and ROS production in microglia [432,433], which have been suggested to play a key role in the pathogenesis of glaucoma [434]. Activated proinflammatory microglia have been implicated in neurotoxicity and RGC phagocytosis and are considered a significant contributor to RGC loss in glaucoma [435]. In contrast, microglia depletion studies using CSF1R blockers show increased RGC loss and progressive astrocytosis using the microbead-induced glaucoma model [436], suggesting a protective role for microglia in glaucoma, highlighting the complexity of the involvement of microglia in glaucoma.

Several preclinical and clinical trials involving antioxidant substances were conducted in glaucoma animal models and clinical trials with promising results: astaxanthin (AST) [437], Vitamin B [401], resveratrol, ascorbyl laurate [438], Ginkgo biloba extract [439], coenzyme Q10 and Vitamin E [440], DHA [441], and many others demonstrate the ameliorative effects of antioxidants on visual functions, retinal blood flow, ERG function, and RGC loss, further establishing glaucoma as an oxidative-stress-mediated pathology. However, while encouraging, none of these antioxidant treatments can fully arrest glaucoma progression.

The pathophysiology of glaucoma is a multifactorial neurodegenerative process affecting multiple cell types in the retina, optic nerve, and TM, leading to complex changes in the microenvironment of the eye. Further investigation of the contribution of various retinal cell types, including neurons and glial cells, and their crosstalk is required to further evaluate the potential of targeting oxidative stress to treat glaucoma or other forms of optic neuropathy. Despite the clear evidence of the prominent role of oxidative stress in the development and progression of POAG, the complexity of POAG pathogenesis has hindered the identification of the precise genes and pathways involved. It is also an obstacle to developing transgenic mouse models that fully recapitulate the pathophysiology of POAG. Further investigations using advanced technologies such as single-cell RNA sequencing open new possibilities for identifying the gene expression signatures of different cell types associated with POAG.

## 6. Targeting Oxidative Stress in Aging

While aging has long been considered a non-modifiable risk factor for many diseases, including those mentioned throughout this review, current trends advocate for the concept that aging itself is a preventable disease [442,443]. Moreover, the processes that underlie resilience and frailty appear to involve many of the same comparative indices that define biological age [444,445,446] and involve the mechanisms in place that promote cellular defenses against oxidative stress, as well as the maintenance of genomic integrity and optimal mitochondrial function [4]. Studies have shown that sera from young mice can promote tissue rejuvenation in older mice, suggesting that factors found in the circulation are important in aging and longevity [447,448]. Consistent with this idea, the activation of conserved adaptive stress responses appears to reduce the pathological effects of aging [449].

Therapeutic interventions to delay age-related degeneration via metabolic regulation have shown promising results in animal models with a focus on longevity as the endpoint [450,451] and are currently under investigation in human clinical trials [452]. An example of modulating bioenergetics to improve physical function has been shown in preclinical studies of the small molecule 1,1-dimethylbiguanide hydrochloride, commonly known as metformin, which is a treatment for type 2 diabetes mellitus and a potent metabolic regulator that promotes improved responsiveness to insulin and aids in stabilizing blood glucose levels [453]. The extended dietary intake of metformin in mice has been found to decrease the expression of markers associated with oxidative damage in the liver (e.g., lysine-4-hydroxinonenal and 8-iso-PGF_2α_), correlating with a ~6% increase in overall lifespan and lower mean body weight [450]. The secondary effects of metformin have been attributed to the activation of the metabolic regulator AMP-activated protein kinase and the potent antioxidant enzyme peroxiredoxin-2 [454]. Metformin has been proposed as a treatment for ocular conditions associated with mitochondrial dysfunction [455,456,457]. Retrospective studies have shown a modest trend of a reduced risk of AMD in subjects taking metformin, though confounding factors such as the presence of cardiovascular disease and diabetes have likely contributed to conflicting findings [458].

At the center of these mechanisms are mitochondrial function and the ubiquitous molecule NAD, a metabolite involved in major metabolic pathways (glycolysis and the TCA cycle, among others) and an important cofactor of the pro-reparative sirtuin enzymes. Preclinical studies have focused heavily on the mitochondrial isoform, SIRT3, an NAD^+^-dependent deacetylase important in the regulation of mitochondrial activity and strongly expressed in highly metabolically active organs, e.g., the heart, liver, kidney, smooth muscle, and brain [459]. Decreased SIRT3 expression in the brain has been associated with Alzheimer’s disease in both human [460] and animal tissues [461], though only small and selective differences in acetylation patterns in human postmortem tissues have been reported [462]. The total or partial loss of SIRT3 in mouse models appears to yield a dramatic phenotype and is associated with hyperacetylation, lower mitochondrial protein expression, and the increased expression of neuroinflammatory markers in the brain [463]. In contrast, the overexpression of SIRT3 in the hippocampi of aged mice appears to decrease neuroinflammation following surgery [464]. Like metformin, NAD supplementation has also been proposed as a small molecule capable of promoting favorable energy utilization and improving overall health [465]. These studies provide a degree of support for the hypothesis that metabolic regulation and mitochondrial integrity are important pathways involved in age-related conditions, which may be modifiable with therapeutic intervention. However, whether these mechanisms can be translated to a decrease in morbidities associated with age-related conditions of the eye remains unclear.

## 7. Conclusions and Future Directions

Redox homeostasis is a delicate balancing act of maintaining appropriate levels of antioxidant defense mechanisms and reactive oxidizing species (ROS and RNS). Any disruption of this balance leads to oxidative stress, which is a key pathogenic factor in several ocular diseases, as highlighted in this review. While excessive reactive oxidizing species are most definitely detrimental to cell function, the same is true of excessive antioxidants. Indeed, physiological levels of ROS and RNS are required for normal cellular function, as they act as second messengers in redox signaling [466]. Second messengers are generated at the instant of receptor activation and act specifically to relay signals to their effectors in a rapid and transient manner. For instance, the mitogen-activated protein kinase (MAPK) family of signaling molecules, including ERK, JNK, and p38, are activated through specific signal transduction cascades that can be activated by H_2_O_2_ [467]. Thus, antioxidant therapies must take into consideration this delicate redox balance and ensure that basal levels of reactive species are maintained for proper cellular functioning.

A myriad of different cell types exists in ocular tissues, each presenting different levels of mitochondrial activity and, thus, different oxidative stress susceptibilities and antioxidant defense mechanisms [468]. For example, mature lens fiber cells in the lens nucleus are devoid of organelles and thus possess no mitochondria and are metabolically quiescent. In contrast, the RPE and photoreceptors are packed with mitochondria to cater to their numerous metabolically demanding functions. While mature lens fiber cells are exposed to less ROS compared to the RPE, they must also depend on the slow, passive diffusion of antioxidants into the central lens nucleus, whereas RPE cells have active organelles to generate a robust antioxidant defense system.

A key unifying risk factor for oxidative stress in ocular diseases is aging. Increased reactive oxidizing species and weakened antioxidant defense systems are associated with increasing age, leading to genetic and epigenetic DNA mutations and subsequent disease. Mitochondria are particularly susceptible to the effects of aging. Metabolic changes and acquired deletions and mutations in mtDNA accumulate over time due to a low replication fidelity, a highly oxidative microenvironment, and the lack of protective histones present in mitochondria compared to the nucleus. These collective changes may contribute to the development of various conditions affecting both the anterior and posterior segments of the eye, as presented in this review (Table 2). Great strides have been made in the fields of oxidative stress, aging, and ocular disease. Continued efforts in this field will not only yield a better understanding of the molecular mechanisms underlying the pathogenesis of ocular diseases but also enable the identification of novel druggable redox targets and antioxidant therapies.

## Figures and Tables

**Table 1:** Summary of the major chemical species associated with oxidative stress in the eye.

Chemical Species	Source(s)	Downstream Reaction(s)	Ref.
Superoxide (O_2_^·−^)	The reaction of O_2_ with enzymes in the electron transport chain in the mitochondria generated via a single-electron transfer; enzymatic and non-enzymatic biosynthetic pathways; produced by neutrophils	Reacts with O_2_^·−^ and H_2_O to generate H_2_O_2_ and O_2_	[13,14,15]
Hydroxyl radical (^·^OH)	The reaction of H_2_O_2_ with iron or copper (Fenton reaction); may also be generated as a byproduct of the exposure of water molecules to ionizing radiation	Reacts with deoxyguanosine residues of DNA to form 8-hydroxy-2-deoxyguanosine; also reacts with deoxycytidine and deoxyadenosine, among others	[16,17]
Hydrogen peroxide (H_2_O_2_)	The reaction of O_2_^·−^ molecules mediated via superoxide dismutase and non-enzymatically; may also be generated as a byproduct of normal catalytic oxidative processes mediated via oxidases	May be converted by myeloperoxidase or other enzymes containing Fe^2+^ or react with UV light to form hydroxyl radical (OH^·^); involved in downstream signaling pathways, such as platelet-derived growth factor signaling	[18,19]
Malondialdehyde (MDA) (CH_2_(CHO)_2_)	Produced by lipid peroxidation of polyunsaturated fatty acids	Reacts with deoxyguanosine of DNA to form 8-hydroxy-2-deoxyguanosine; also reacts with deoxyadenosine residues; may also react with lysine residues on proteins to form secondary oxidation products	[20,21]
4-Hydroxynonenal (4-HNE) (CH_3_(CH_2_)_4_CH(OH)CH=CH(CHO))	Produced by lipid peroxidation of polyunsaturated fatty acids or linoleic or arachidonic side chains	Reacts with lysine on proteins to form carbonylated side chains, increasing the hydrophobicity of modified proteins; also involved in downstream signaling pathways, including activation of glutamate-cysteine ligase expression	[22,23]

**Table 2:** Summary of key findings associating oxidative stress with pathologies affecting the anterior and posterior segments of the eye (Abbreviations: electron transport chain (ETC); reduced glutathione (GSH); 4-hydroxynonenal (4-HNE); malondialdehyde (MDA); nuclear factor-erythroid 2 related factor 2 (NRF2); 8-hydroxy-2-deoxyguanosine (8-OHdG); retinal ganglion cells (RGCs); retinal pigment epithelium (RPE); superoxide dismutase (SOD); tricarboxylic acid (TCA)).

Condition	Clinical Manifestation(s)	Identified Markers of Oxidative Stress	Ref.
Dry eye disease	Instability of the lipid layer, decreased tear secretion, ocular irritation	Elevated 8-OHdG, 4-HNE, and MDA and higher immune infiltration in dry eye animal models; elevated 4-HNE and hexanoyl-lysine in the conjunctiva of patients with dry eye and Sjögren’s syndrome	[42,43,45]
Keratoconus	Thinning of the corneal stroma leading to bulging of the central cornea	Elevated lipid peroxidation, MDA, and proinflammatory cytokines in tears of patients with keratoconus; increased lactate production and altered glycolytic and TCA cycle metabolite levels in corneal fibroblasts; decreased NRF2 expression	[64,65,66,67]
Cataract	Opacification of the lens	Oxidation, deamidation, and other chemical modifications of crystallin proteins, leading to protein aggregation and precipitation; decreased GSH in lens epithelial cells	[117,121,138]
Age-related macular degeneration	Drusen formation and photoreceptor degeneration; choroidal neovascularization, hemorrhage, and retinal fibrosis	Elevated MDA and 8-OHdG; decreased SOD in RPE cells; elevated carboxyethylpyrrole in drusen	[221,225,233]
Proliferative vitreoretinopathy	Formation of fibrotic membranes on the retinal surface	Reduced SOD and catalase in vitreous; decreased GSH in vitreous and blood	[283,284]
Diabetic retinopathy	Vascular abnormalities in the retina; microaneurysms, hemorrhaging, and angiogenesis	Increased ROS production by ETC with hyperglycemia; decreased SOD, catalase, and GSH production	[321,322,329]
Glaucoma	Loss of RGCs in the retina	Reduced total reactive antioxidant potential and increased MDA in the aqueous humor and blood	[381,382,424]
